# Biohybrid CO_2_ electrolysis under external mode: Using pure formic acid extracted from CO_2_ electroreduction for diverse microbial conversion

**DOI:** 10.1016/j.fmre.2024.02.008

**Published:** 2024-03-05

**Authors:** Na Chu, Xiaobing Wu, Ziyue Zhao, Xue Zheng, Yilin Lu, Ying Pu, Yue Wang, Jiayi Cai, Lixia Zhang, Xiaohong He, Daping Li, Raymond Jianxiong Zeng, Yangyang Yu, Yong Jiang

**Affiliations:** a CAS Key Laboratory of Environmental and Applied Microbiology, Environmental Microbiology Key Laboratory of Sichuan Province, Chengdu Institute of Biology, Chinese Academy of Sciences, Chengdu 610041, China; b University of Chinese Academy of Sciences, Beijing 100049, China; c Fujian Provincial Key Laboratory of Soil Environmental Health and Regulation, College of Resources and Environment, Fujian Agriculture and Forestry University, Fuzhou 350002, China; d School of Life Science, Beijing Institute of Technology, Beijing 100081, China; e Information Materials and Intelligent Sensing Laboratory of Anhui Province, Institutes of Physical Science and Information Technology, Anhui University, Hefei 230601, China

**Keywords:** Carbon dioxide reduction, Microbial electrosynthesis, Gas diffusion electrodes, Flow cell, Renewable energy storage

## Abstract

The demand for converting CO_2_ into fuels or chemicals is on the rise to achieve a carbon-efficient circular economy. Biohybrid CO_2_ electrolysis shows potential for increasing production rates and diversifying product spectra by combining electrocatalysts and microbial catalysts. However, it is important to note that utilizing a shared catholyte for biohybrid CO_2_ electrolysis has not demonstrated significant performance improvements to date. In this study, we developed a biohybrid CO_2_ electrolysis system utilizing a solid electrolyte operating in an external mode. The produced formic acid was extracted and used as an intermediate for microbial conversion. Impressively, the solid-electrolyte CO_2_ electrolysers obtained a remarkable total Faradic efficiency of 81.4% for formic acid production. *In-situ* mechanism studies unveiled metallic tin as the probable real active site, prompting further exploration of strategies to boost the activity and stability of electrocatalysts. In the bioconversion step, we achieved a noteworthy 8-day duration for generating bioelectricity, nearly 100% electron recovery for biomethane production, and 90.8% for acetate generation. Additionally, when ethanol was co-fed, a C_6_ specificity of 41.1% was observed for the generation of medium-chain fatty acids (MCFAs). This study presents groundbreaking experimental data that demonstrates the numerous advantages of utilizing hybrid systems as advanced synthesis techniques.

## Highlights

• Biohybrid CO_2_ electrolysis was created with electrocatalysts/microbial catalysts.
• Formic acid was extracted as the intermediate for bioconversion.
• Total Faraday efficiency of formic acid was 78.4 ± 3.0% at 60 mA cm^2^.
• Bioelectricity, biomethane, acetate, and MCFAs were generated in bioconversion.
• External mode supported a high current density and a diverse microbial conversion.

## Graphical abstract

**Figure:**
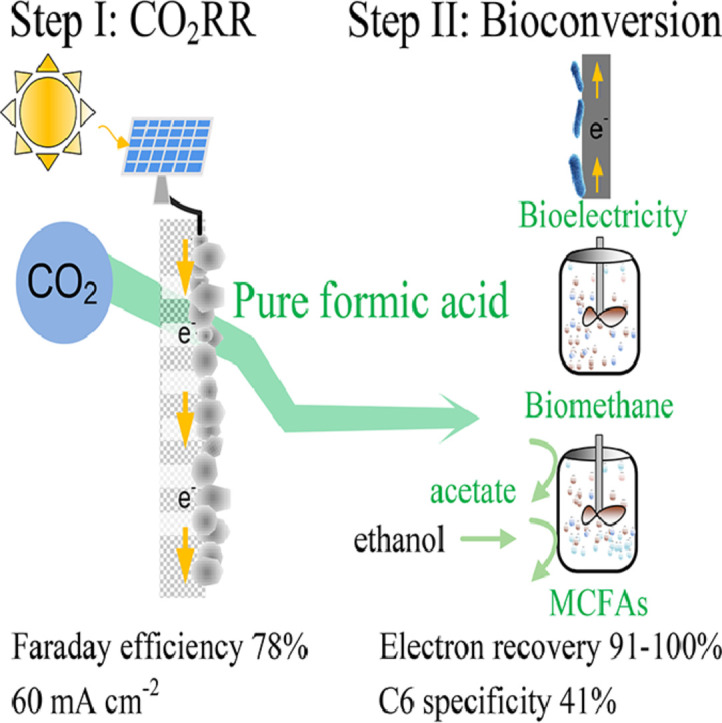

## 1 Introduction

The synthesis of fuels and chemicals from CO_2_ has emerged as a highly promising strategy to address the urgent global challenges of climate change and petroleum depletion [1,2]. In this context, the catalytic reduction of CO_2_, employing electrocatalysts and/or microbial catalysts, has gained significant attention in the energy and environmental fields [3,4].

Electrocatalytic reduction of CO_2_ (CO_2_RR) has proven effective in generating C_1_ compounds (CO and formate) with both high Faradic efficiency and current density [5,6]. However, the progress in C_2+_ compound generation from CO_2_RR has not mirrored the advancements seen in C_1_ production over the last decade [7]. Previous studies have shown that the Faradic efficiency for producing acetate and methane tends to be below 70%, at current densities typically less than 10 mA cm^−2^ [8,9]. In a recent investigation, even with the utilization of advanced noble-metal-based electrocatalysts, the Faradic efficiency for generating C_6_ chemicals from CO_2_RR was only 0.16% at a current density of 14 mA cm^−2^ [10].

The microbial electrochemical system stands out as a versatile platform technology with multifaceted functionalities. Originally centered on producing electricity by breaking down pollutants through microbial fuel cells (MFC), recent investigations have revealed its potential for fuel and chemical production through processes like CO_2_ reduction and organic waste valorization, collectively known as microbial electrosynthesis (MES). In MES, highly reduced C_1_ (methane) and C_2_ (acetate) compounds can be produced from CO_2_ with a remarkable Faradic efficiency approaching 100%, owing to the exceptional selectivity of microbial catalysts [11,12]. Stability tests conducted over a year attest to the longevity of MES, attributed to the self-renewal capability of microbial catalysts [11,^13^,14]. This contrasts with electrocatalysts, which often exhibit stability only within the initial few hours, though the catalytic activity of microbial catalysts tends to be lower, resulting in a current density typically below 2 mA cm^−2^ [12], at least one order of magnitude lower than that of CO_2_RR.

Biohybrid CO_2_ electrolysis holds the promise of boosting production rates by leveraging electrocatalysts while simultaneously broadening the product spectrum through the use of microbial catalysts [15,16]. In the initial demonstration of this concept, operating in an internal mode, an indium (In) foil cathode was used to generate formate as an intermediate product. Engineered *Ralstonia eutropha* was then employed to convert formate into isobutanol and 3-methyl-1-butanol [17]. Formate was selected as the intermediate due to its ability to overcome gas-liquid mass transfer challenges associated with CO, and its advantage of easy storage and transport. However, previous studies have indicated that the performance of biohybrid CO_2_ electrolysis did not significantly improve when electrocatalysts and microbial catalysts were utilized in the conventional internal mode, coexisting in a single vessel with a shared catholyte [18].

The internal mode of biohybrid CO_2_ electrolysis poses challenges by potentially suppressing both electrocatalysts and microbial catalysts. To mitigate the hydrogen evolution reaction (HER) during CO_2_RR, a concentration of 1 M or higher of KOH or KHCO_3_ is typically used. However, the employment of such high salt and hydroxyl ion concentrations may lead to cell damage. For example, the use of acetate dissolved in 1 M KOH or KHCO_3_ as an intermediate has been shown to impede the growth of *Chlamydomonas*, resulting in a 50% decrease in biomass [19]. Furthermore, reagents in microbial culture media preparation, such as yeast extraction, phosphate buffer solution (PBS), and other impurities, can contribute to the deterioration of electrocatalysts [12].

Biohybrid CO_2_ electrolysis under external mode has the potential to optimize CO_2_RR and bioconversion individually. However, a challenge similar to the one encountered in the case of internal mode arises when utilizing intermediates dissolved in highly concentrated electrolytes for microbial culture [20]. Separating intermediates from the high electrolyte concentration may require costly processes such as electrodialysis or distillation [21]. Alternatively, sustaining a high intermediate-to-salt ratio enables the proper dilution of the catholyte before the bioconversion step [19].

Due to these limitations, the implementation of biohybrid CO_2_ electrolysis has been somewhat restricted, particularly when both electrocatalysts and microbial catalysts share a catholyte or when intermediates are dissolved in a highly concentrated electrolyte. Recently, the direct production of pure formic acid from CO_2_RR has been considered a key advancement in this field [22], potentially opening new avenues for advanced synthesis techniques.

In this study, we introduced a pioneering technique termed biohybrid CO_2_ electrolysis under external mode. This innovative approach streamlines the extraction of pure formic acid from a novel solid-electrolyte CO_2_ electrolysers. Furthermore, the study explored the diverse applications of this technology, assessing bioelectricity generation for energy storage, biomethane production as a gaseous biofuel, and the synthesis of acetate and medium-chain fatty acids (MCFAs) as valuable liquid biochemicals in the bioconversion step. To deepen our comprehension of the process, we conducted a systematic characterization of the gas diffusion electrode (GDE) and analyzed the enriched microbial communities. By offering these insights, this research makes a significant contribution to maximizing the environmental and energy potential of biohybrid CO_2_ electrolysis.

## 2 Materials and methods

### 2.1 Construction and operation of solid-electrolyte CO_2_ electrolysers

**Fig. 1:**
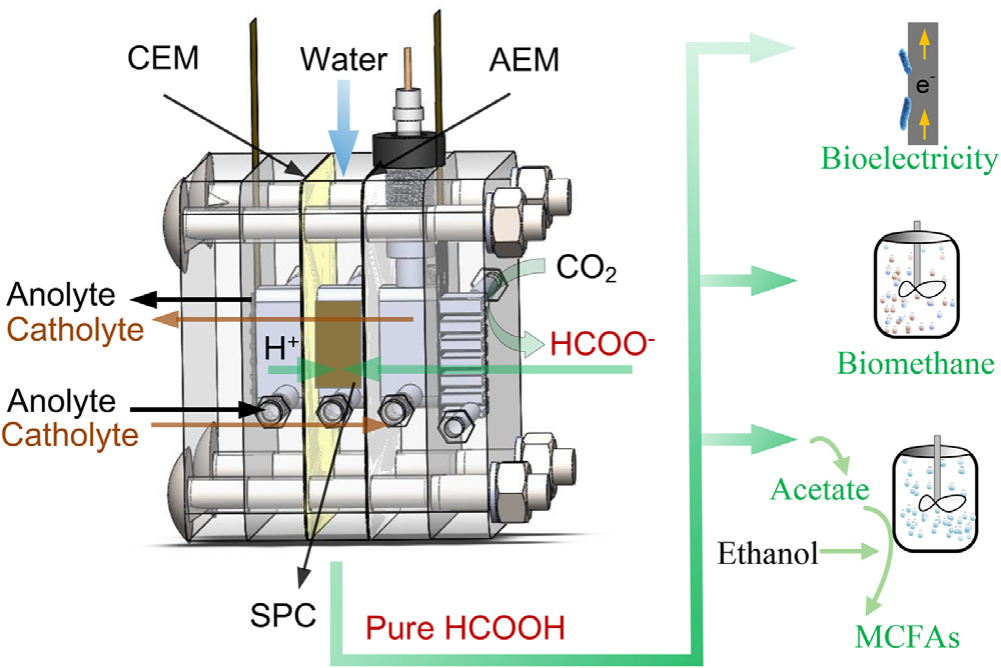
**Biohybrid CO_2_ electrolysis under external mode: using pure formic acid extracted from solid-electrolyte CO_2_RR for diverse microbial conversion**.

Solid-electrolyte CO_2_ electrolysers, configured as four-chamber flow cells, were employed for the production of pure formic acid (Fig. 1 and Fig. S1). The anode featured a platinum mesh (2 × 1.5 cm^2^), while the cathode consisted of an airbrushed SnO_2_/C GDE. The catalyst ink for the cathode was prepared by combining commercial SnO_2_ nanoparticles (50–70 nm, Macklin, Shanghai, China) and carbon black (Vulcan XC 72R, Cabot) in an optimized mass ratio of 3.5, with a total mass of 20 mg. This mixture was then blended with 267 μL of isopropanol and 88 μL of Nafion ionomer solution (D520, 5.0 wt%, Heshen Co., Shanghai, China), resulting in a homogeneous catalyst ink [23]. The ink was airbrushed onto 2 × 1 cm^2^ commercial gas diffusion layers (GDL) (Sigracet 29 BC, SGL Group, USA), resulting in a SnO_2_/C GDE with a catalyst and carbon black loading of approximately 4 mg cm^−2^. An Ag/AgCl electrode (Gaoss Union Co., Ltd., Wuhan, China) was inserted into the cathode chamber. The anode, cathode, and extraction chambers were each constructed from 1-cm-thick polymethyl methacrylate with 1-cm-wide by 2-cm-long channels, separated by a pair of ion exchange membranes (TWEDC1 and TWEDA1, Shandong Tianwei Membrane Technology Co., Ltd, China). The chambers were connected to 120 mL circulating bottles, with each bottle containing 50 mL of anolyte, catholyte, and deionized water, respectively. In the gas chamber, CO_2_ (> 99.9%) was continuously supplied to the backside of the SnO_2_/C GDE at a fixed rate of 20 mL min^−1^. The anolyte was 0.5 M H_2_SO_4_, the catholyte was 1 M KOH, and the extraction chamber contained porous styrene–divinylbenzene copolymer (AmberChrom® 50WX2, 200–400 mesh) as the solid proton conductor (SPC) [24]. This configuration allowed H^+^ to migrate from the anode chamber into the extraction chamber, while HCOO^−^ produced from CO_2_RR could migrate from the cathode chamber into the extraction chamber. Consequently, pure formic acid was formed and could be extracted from the solid-electrolyte using deionized water.

The effects of current density and the flow rate of deionized water were evaluated. Different current densities (30, 40, 50, and 60 mA cm^−2^, corresponding to 60, 80, 100, and 120 mA) were applied to CO_2_ electrolysers, while the flow rate of deionized water, anolyte, and catholyte were all fixed at 2 mL min^−1^. Additionally, different flow rates of deionized water in the extraction chamber (0.5, 2, and 10 mL min^−1^) were adjusted, while the current density was fixed at 60 mA cm^−2^, and the flow rate of anolyte as well as catholyte were kept at 2 mL min^−1^.

As a control test, a four-chamber flow cell without SPC was used for CO_2_RR, and the extraction chamber was filled with 1 M KOH. Furthermore, an additional control test was conducted with a conventional three-chamber flow cell, where a piece of anion exchange membrane (AEM) was directly used to cover the GDE. All electrochemical measurements were conducted in duplicate at room temperature. Additionally, a membrane electrode assembly (MEA) based solid-electrolyte CO_2_ electrolysers (4 cm^2^) was constructed to further enhance energy efficiency (Fig. S2).

### 2.2 Bioconversion step using pure formic acid as the feedstock

Pure formic acid was obtained from CO_2_RR by operating solid-electrolyte CO_2_ electrolysers at a constant current of 60 mA cm^−2^ for 5 h, with a fixed flow rate of 2 mL min^−1^ for deionized water, anolyte, and catholyte. All bioconversion tests were conducted in duplicate. Multiple runs of the solid-electrolyte CO_2_ electrolysers were performed to ensure that the formic acid used for bioconversion indeed originated from CO_2_RR. The purity of the collected formic acid was systematically verified using inductively coupled plasma optical emission spectrometry (ICP-OES, Avio 200, PerkinElmer Inc.,Waltham, MA, USA), pH, and conductivity measurements.

For bioelectricity generation using the collected pure formic acid as feedstock, microbial 3-electrode setups with a total volume of 50 mL were assembled. The counter electrode consisted of a piece of platinum sheet (2 cm × 2 cm × 0.01 cm). The working electrode was a circular piece of carbon felt (diameter 2.2 cm, thickness 0.5 cm), and the anodic biofilms were initially enriched in a two-chamber MFC in our laboratory, fed with formic acid as the carbon and energy source (Fig. S3). The reference electrode was made of Ag/AgCl (Gaoss Union Co., Ltd., Wuhan, China). Microbial 3-electrode setups were initially filled with 40 mL of formic acid solution prepared using analytical reagents, amended with 0.8 mL of concentrated nutrient medium. The composition of the final medium was as follows: formic acid, ∼45 mM; K_2_HPO_4_, 2.6 g L^−1^; KH_2_PO_4_, 4.4 g L^−1^; NH_4_Cl, 0.31 g L^−1^; MgCl_2_•6H_2_O, 0.2 g L^−1^; Na_2_SO_4_, 0.05 g L^−1^; trace elements, 12.5 mL; and vitamin solution, 5 mL [25]. The initial pH was adjusted to 7, and the working electrode was poised at +0.1 V versus the standard hydrogen electrode (SHE) [26]. Upon the current dropping to near 0 mA, microbial 3-electrode setups were emptied and filled with fresh medium, where the 40 mL of formic acid solution was collected from CO_2_RR. These microbial 3-electrode setups were operated at room temperature without magnetic stirring.

For biomethane generation using the collected pure formic acid as feedstock, serum vials with a total volume of 115 mL were employed. The medium was prepared with 45 mL of formic acid solution collected from CO_2_RR and amended with the aforementioned 0.9 mL of concentrated nutrient medium. The concentration of formic acid in the prepared medium was ∼45 mM. Anaerobic sludge was cleaned with 50 mM PBS three times before being used as the inoculation. These serum vials were incubated at 30 °C in a 120-rpm shaker after adjusting the initial pH of the medium to 7.

For acetate and MCFAs generation using the collected pure formic acid as feedstock, serum vials with a total volume of 115 mL were utilized. An additional adaptation period was required when using formic acid as the electron acceptor for MCFAs production [27]. The initial inoculation involved anaerobic sludge, and the adaptation period lasted 85 days (Fig. S4). Enriched microbes were cleaned with 50 mM PBS three times before being used as the final inoculation. Three groups were created: Group FAET (Formic Acid and Ethanol Co-Feeding) was added with both formic acid and ethanol in a molar ratio of 1:4, while the total carbon content was fixed at 400 mM C [28]. These serum vials were filled with 45 mL formic acid solution collected from CO_2_ electrolysers and amended with 0.9 mL concentrated nutrient medium. The composition of the final medium was as follows: formic acid, ∼45 mM; ethanol, ∼180 mM; K_2_HPO_4_, 2.6 g L^−1^; KH_2_PO_4_, 4.4 g L^−1^; NH_4_Cl, 0.31 g L^−1^; MgCl_2_•6H_2_O, 0.2 g L^−1^; Na_2_SO_4_, 0.05 g L^−1^; 2-BES, 4.22 g L^−1^; trace elements, 12.5 mL; and vitamin solution, 5 mL. Group FA (Formic Acid-Feeding only) was only added with the electron acceptor (formic acid), while Group ET (Ethanol-Feeding only) was only added with the electron donor (ethanol). These serum vials were incubated at 30 °C in a 120-rpm shaker after adjusting the initial pH of the medium to 7.

### 2.3 Analyses and calculations

Formic acid and caproic acid were analyzed using high-performance liquid chromatography (HPLC, LC-2030, Shimadzu Scientific Instruments Inc., Kyoto, Japan), while other organic acids and alcohols were detected using gas chromatography (GC-2030, Shimadzu Scientific Instruments Inc., Kyoto, Japan) [14,29]. Additionally, formic acid-¹³C (95 wt.% in H₂O, Aladdin, Shanghai, China) was employed as the electron acceptor during MCFAs generation, and the isotope samples were analyzed using various techniques, including ^1^H-nuclear magnetic resonance (NMR, Bruker Avance NEO 400 MHz, Germany), ^13^C-NMR, and mass spectrometry (Thermofisher Exactive Plus, America) [30].

The contents of CH_4_ and H_2_ were analyzed using another gas chromatography analyzer (SP 6890, Lunan Inc., Tengzhou, China). CO_2_ electrolysers operated in the galvanostatic mode, with the current and voltage automatically recorded using a battery testing system (CT-4008; Neware Technology Co., Ltd., Shenzhen, China) (CT-4008; Neware Technology Co., Ltd., Shenzhen, China) [31]. Microbial 3-electrode setups were operated under potentiostatic control, with a potential fixed at +0.1 V versus SHE by an electrochemical workstation (CS3106, Corrtest Instruments Corp. Ltd., Wuhan, China). Dissolved metal contents were measured by ICP-OES [18], and conductivity was measured using an online conductivity meter (SevenExcellence, Mettler Toledo, Switzerland). Microbial diversity of enriched microbes was performed on a commercial platform (Majorbio Bio-pharm Technology Co., Ltd., Shanghai, China) after DNA extraction, PCR, and DNA sequencing [17,18]. Bacteria analysis used the 338F/806R primers, while archaea analysis used the 524F10extF/Arch958R primers.

The surface morphology of the SnO_2_/C GDE was examined using a scanning electron microscope (SEM, Hitachi SU8020, Japan), and the crystal structure of the catalyst surface was evaluated by X-ray diffraction (XRD, Brucker D8 Advance, Germany). The hydrophobicity of the SnO_2_/C GDE was tested using a contact angle measurement instrument (JC2000d, PowerEach, China).

Linear sweep voltammetry (LSV) analysis of the CO_2_ electrolysers before and after operation was performed using an electrochemical workstation (CHI 660E, Chenhua Instrument Co., Shanghai, China), with a scan range from 0 to −1.2 V versus RHE and a scan rate of 10 mV s^−1^. Double-layer capacitance (*C*_dl_) and electrochemical surface areas (ECSA) of the SnO_2_/C GDE before and after operation were evaluated by cyclic voltammetry (CV). The scanning range was from 0.6 to 0.5 V versus RHE, and the scanning rate was from 20 to 200 mV s^−1^. Faradic efficiency of formic acid generation in the CO_2_ electrolysers and Coulombic efficiency of bioelectricity generation in the microbial 3-electrode setup were calculated considering the molar conversion factor (Text S1) [23].

*In-situ* Raman was performed using a Raman spectrum (LabRam HR, Horiba, France) with a 633 or 785 nm laser for excitation. To enhance signal intensity during t-mapping test, the shell-isolated nanoparticle enhanced Raman spectroscopy (SHINERS) was introduced according to a previous study [32]. The electrolyte was 1 M KOH purged with CO_2_, and the tests were conducted from −0.2 to −2.4 vs. RHE with the recording of results under open-circuit potential (OCP). *In-situ* attenuated total reflectance infrared (ATR-IR) spectroscopy was conducted by an INVENIO-R infrared spectrometer (Bruker, Germany), according to a previous study [33].

The carbon balance and reduction degree balance during biomethane production were calculated to confirm the selectivity of the bioconversion step and the reliability of the experimental data for product analysis. Note that the carbon balance was calculated based on the carbon content per mole of chemicals (e.g., 2 mol carbon for 1 mol acetate), and the reduction degree was calculated based on the corresponding molar conversion factor of chemicals (e.g., 8 for 1 mol acetate). The C_6_ specificity during MCFAs production was calculated based on the electron recovery in caproic acid, compared with all identified liquid products [13]. Full-cell energy efficiency was calculated based on the energy input of the electricity and the standard heat of combustion of chemical substances (Text S1).

## 3 Results and discussion

### 3.1 Pure formic acid production from CO_2_RR

**Fig. 2:**
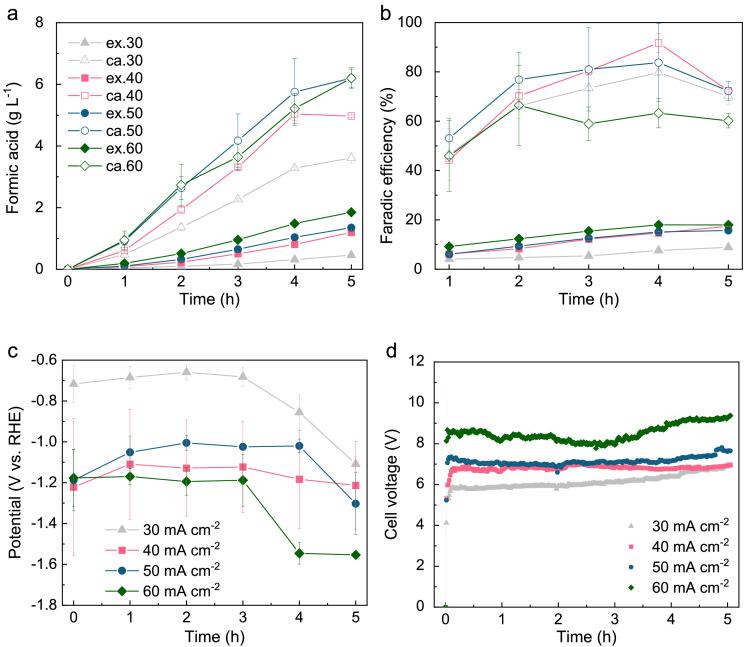
**Effects of current density on CO_2_RR**. (a) Formic acid concentration, (b) Faradic efficiency, (c) cathode potential, and (d) cell voltage. Abbreviation: an., anode; ex., extraction; and ca., cathode. Marked numbers were current densities.

Various current densities were applied to the solid-electrolyte CO_2_ electrolysers (Fig. 2). At a current density of 60 mA cm^−2^, formic acid concentrations reached 6.2 ± 0.3 g L^−1^ (cathode chamber) and 1.8 ± 0.1 g L^−1^ (extraction chamber) after 5 h. The accumulation of formic acid in the anode chamber increased with the current density, but the final concentration remained below 0.03 g L^−1^. The total Faradic efficiency of formic acid (sum of all chambers) reached 81.4% at the 4th hour. A decrease in current density resulted in lower formic acid concentrations, an increase in cathode potential, and a decrease in cell voltage. Surprisingly, the highest total Faradic efficiency of formic acid was achieved at a current density of 40 mA cm^−2^, reaching nearly 100% at the 4th hour but dropping to about 90% at the 5th hour. It is noteworthy that the applied current density in this study falls within a similar range to other studies on solid-electrolyte CO_2_ electrolysers [22,34], and is at least one order of magnitude higher than that of previous studies on MES [35,36]. The Faradic efficiency calculated at the 1st hour aligns with a previous study on SnO_2_/C GDE [23]. However, the time course of Faradic efficiency suggests that the activity of SnO_2_/C initially increased but subsequently decreased during operation. The activity and stability of SnO_2_ can be significantly influenced by the potential-pH combination. Notably, a slight decrease in pH in the catholyte was observed during operation (Fig. S5). Additionally, a sharp negative shift in cathode potential, coupled with an increase in cell voltage, was observed after the 3rd hour. These findings suggest that the decrease in Faradic efficiency could be attributed to the degradation of the electrocatalyst, a challenge that has been identified as a major issue in CO_2_RR [22,37].

The flow rate of deionized water in the extraction chamber demonstrated no impact on the accumulation and distribution of formic acid (Fig. S6), nor did it influence Faradic efficiency or pH evolution (Fig. S7). These observations diverge from prior studies where deionized water in the extraction chamber was flown in a single-pass mode [24,38]. In this study, a circulating bottle was employed to enhance formic acid accumulation during the extraction process using deionized water. Consequently, it was unexpected that the flow rate of deionized water would exert a substantial influence on formic acid concentration within the packed SPC layer, thereby affecting reverse reactions.

To gain a more comprehensive understanding of the energy losses in solid-electrolyte CO_2_ electrolysers, two control tests were conducted. A conventional GDE-equipped three-chamber flow cell was utilized, yielding a cell voltage of 5.2 ± 0.1 V. Notably, neither a solid electrolyte nor an extraction chamber was employed. This voltage was only half of that observed in the solid-electrolyte CO_2_ electrolysers and remained remarkably stable throughout the operation (Fig. S8). Additionally, the formic acid accumulation and Faradic efficiency obtained from this conventional three-chamber flow cell were consistent with findings from previous studies [23]. When the four-chamber flow cell was filled with a 1 M KOH solution (without SPC) as an alternative in the extraction chamber, the cell voltage was observed to be approximately halved (Fig. S9). These results suggest that the addition of an extra extraction chamber only marginally increases the cell voltage, while the inclusion of SPC significantly amplifies the energy consumption.

### 3.2 Ultrahigh purity of the collected formic acid solution

**Fig. 3:**
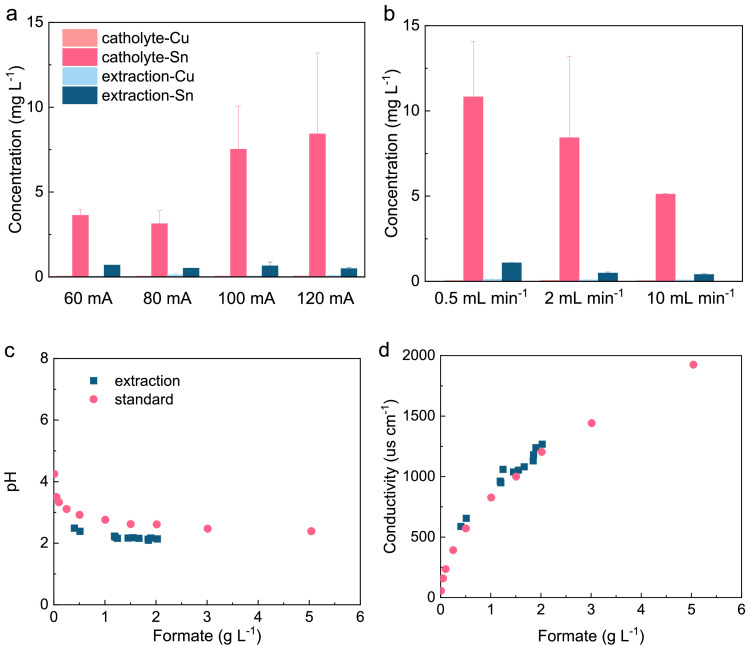
**Demonstration the ultrahigh purity of the formic acid solution collected from CO_2_RR.** Tin and copper contents at different (a) current densities and (b) flow rates of the extraction solution. Comparation of (c) pH and (d) conductivity between the formic acid solution collected from CO_2_RR and standard solution prepared with analytical reagents.

A transparent formic acid solution was obtained through CO_2_RR (Fig. S10). Its purity was comprehensively assessed through ICP-OES, pH, and conductivity measurements (Fig. 3). Notably, the obtained formic acid solution still contained some residual salt. Specifically, the tin content in the collected formic acid solution (extraction solution) was approximately ten times lower than that in the catholyte. At a flow rate of 0.5 mL min^−1^ and a current density of 60 mA cm^−2^, the tin content in the extraction solution reached its maximum value of 1.09 ± 0.02 mg L^−1^. Interestingly, the tin content in the catholyte increased with the current density. However, a reverse trend was observed when the flow rate of deionized water was changed. Tin potentially released from the SnO_2_/C GDE, the instability of which has been reported in previous studies [23]. It is crucial to highlight that tin demonstrates excellent biocompatibility. For instance, researchers have utilized SnO_2_ nanocomposites to decorate anodes, facilitating stimulated biofilm attachment and bioelectricity generation [39]. Copper could potentially be released from the degradation of copper foil tape, but its concentration remained below 0.1 mg L^−1^ in both the catholyte and extraction solution. On the other hand, the extraction solution showed a considerable accumulation of potassium, reaching levels as high as 7.82 ± 0.02 mg L^−1^ when the flow rate was set to 10 mL min^−1^. The concentrations of other ions, such as sodium, iron, bismuth, and silver, generally remained below 1.0 mg L^−1^ (Table S1). Previous studies on solid-electrolyte CO_2_ electrolysers have noted that the concentration of impurity ions can vary significantly, ranging from as low as 10 ppb to as high as 100 ppm [24,40].

The pH of the formic acid solution generated through CO_2_RR ranged from 2.1 to 2.5, only slightly lower compared to solutions prepared using analytical reagents. Moreover, the conductivity of the formic acid solution produced from CO_2_RR closely matched that of solutions prepared with analytical reagents. Considering the results obtained from ICP-OES analysis, pH measurements, and conductivity tests, it becomes evident that the formic acid solution collected from CO_2_RR demonstrates an exceptionally high level of purity.

### 3.3 Characterization of SnO_2_/C GDE before and after CO_2_RR

**Fig. 4:**
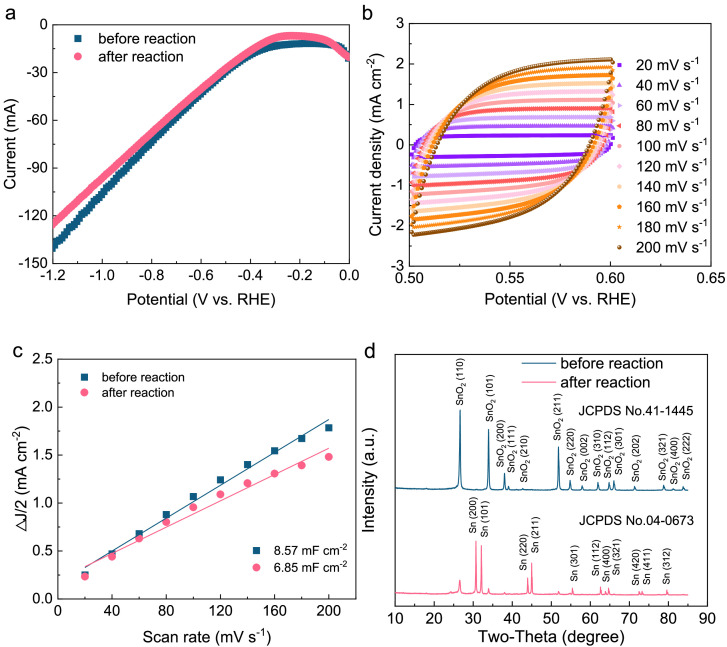
**Characterization of SnO_2_/C GDE before and after the CO_2_RR with a current density fixed at 60mA cm^−2^.** (a) LSV analysis of the CO_2_ electrolysers, (b) CV curves over a range of scan rates, (c) *C*_dl_ calculation, and (d) XRD spectra of SnO_2_/C GDE.

LSV curves of the solid-electrolyte CO_2_ electrolysers were conducted (Fig. 4a). Prior to electroreduction (using a freshly prepared electrode), a current density of 107 mA cm^–2^ was achieved at −1.0 V versus RHE. However, after operation, the current density slightly decreased to 97 mA cm^–2^. In a previous study [41], it was found that the activity of the SnO_2_/C GDE was significantly lower compared to newly fabricated ones. The CV curves of the SnO_2_/C GDE (Fig. 4b and Fig. S11) indicated that the *C*_dl_ before electroreduction was determined to be 8.57 mF cm^–2^. However, after electroreduction, it decreased to 6.85 mF cm^–2^ (Fig. 4c). The relatively high values of the calculated *C*_dl_ could be attributed to the use of capacitive carbon black in the system. Furthermore, the decrease in capacitance characteristics after electroreduction suggests the possibility of partial shedding of the catalyst.

The XRD spectra indicated a shift from polycrystalline SnO_2_ to polycrystalline Sn after the CO_2_RR (Fig. 4d). This change aligned nicely with the observed surface morphology. Initially, a homogeneous combination of SnO_2_ particles (50–70 nm) and carbon black particles (∼50 nm) was coated onto the GDE. However, following the CO_2_RR, the formation of polycrystalline Sn ranging from 200 to 500 nm was observed (Fig. S12). The transition from a contact angle of 153.8 to 91.5 indicates an enhanced hydrophilic nature following CO_2_RR. This increased hydrophilicity contributed to the potential of GDE flooding [22,37]. Interestingly, even at a potential lower than that required for CO_2_RR, the reduction of SnO_2_ to Sn can occur [23]. Additionally, a decrease in pH can further intensify the instability of SnO_2_. The long-term stability of the SnO_2_/C GDE was assessed. During this evaluation, formic acid was produced with a commendable Faradic efficiency of approximately 75%. Notably, there was minimal decay observed within the first 10 h of continuous operation. However, an evident decline in Faradic efficiency was observed when the operation time was extended beyond that (Fig. S13).

### 3.4 Bioelectricity generation using pure formic acid generated from CO_2_RR

**Fig. 5:**
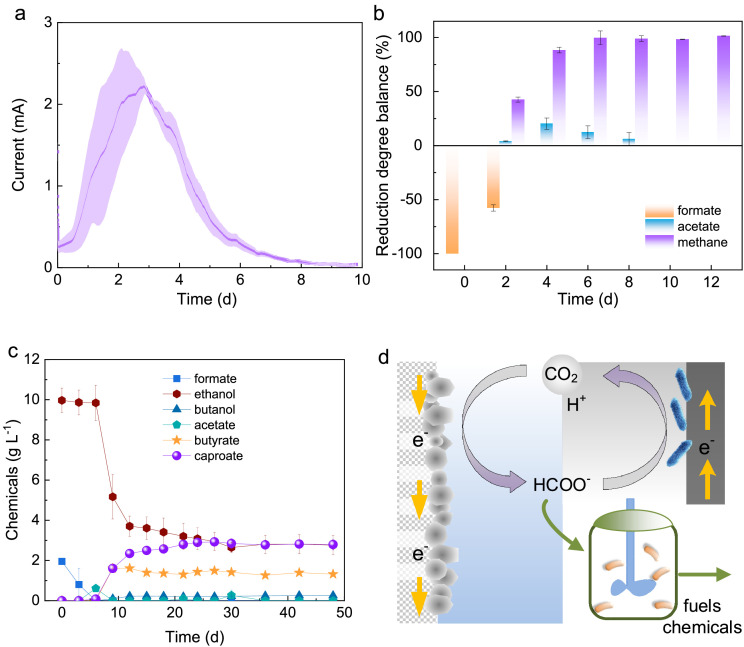
**Bioconversion step using pure formic acid generated from CO_2_RR as the feedstock.** (a) Time course of current output in microbial 3-electrode setups. (b) Time course of reduction degree balance for biomethane generation. (c) MCFAs generation in the FAET group. (d) Schematic diagram of using pure formic acid as the feedstock for bioelectricity generation to close the carbon cycle and achieve CO_2_-to-fuels or chemicals conversion.

Microbial 3-electrode setups were utilized for bioelectricity generation using pure formic acid obtained from CO_2_RR. By setting the working electrode at a fixed potential of +0.1 V versus SHE, a peak current density of 0.58 ± 0.03 mA cm^−2^ was achieved (Fig. 5a). This trend remained consistent when the formic acid-containing medium was prepared using analytical reagents as well (Fig. S14). Notably, a remarkable bioelectricity recovery period of 8 days was observed, making it particularly suitable for various applications such as wireless sensor networks and water biotoxicity monitoring. The microbial consumption of formic acid was confirmed through HPLC analysis.

### 3.5 Biomethane generation using pure formic acid generated from CO_2_RR

To convert CO_2_ into gaseous biofuels, the viability of microbial-based methanogenesis was evaluated using pure formic acid produced from CO_2_RR (Fig. S15). The complete consumption of formic acid was observed within a span of 4 days, and subsequently, methane accumulation reached a stable level after 8 days. Throughout the biomethane generation process, traces of H_2_ (below 0.6 mL) and acetic acid (below 0.3 g L^−1^) were detected but eventually disappeared by the end of the operation. This suggests that H_2_ and acetic acid act as intermediates during the bioconversion of formic acid, consistent with findings from a previous study [42].

The carbon distribution and reduction degree balance (Fig. 5b) were analyzed, resulting in a carbon recovery rate of 25.4 ± 0.1%. Additionally, the reduction degree balance yielded relative errors of less than 2%. It is noteworthy that formic acid, with its capacity to carry only 2 electrons per carbon, is not highly reduced for many applications. Consequently, when formic acid is employed as the feedstock for biomethane generation, approximately 3/4ths of the carbon will theoretically be released back into the atmosphere [43].

### 3.6 Acetate and MCFAs generation using pure formic acid generated from CO_2_RR

To convert CO_2_ into liquid biochemicals, the Formic Acid and Ethanol Co-Feeding (FAET) process was employed, utilizing formic acid as the electron acceptor and ethanol as the electron donor for microbial chain elongation (Fig. 5c). The production of butyric acid and caproic acid exhibited a lag phase of 6 days. Ultimately, the concentration of butyric acid reached 1.3 ± 0.1 g L^−1^, while caproic acid achieved a concentration of 2.8 ± 0.3 g L^−1^. The C_6_ specificity was found to be 41.1 ± 0.2%. This value is significantly higher compared to previous studies in MES, where the electrode served as the sole electron donor and CO_2_ as the sole carbon source [13]. Interestingly, in the Formic Acid (FA) group (Fig. S16), no caproic acid was produced; instead, acetate was the main product with an electron recovery rate of 90.8%. On the other hand, in the Ethanol (ET) group, the concentration of caproic acid was below 0.25 g L^−1^, with only a small amount of butyric acid detected as well. It is important to note that the direct production of C_4+_ chemicals from CO_2_RR is a challenging process and has been rarely reported [12]. In contrast, microbial CE platforms have shown the ability to biosynthesize MCFAs with high specificity, provided that an optimized ratio of electron acceptors and electron donors is provided [13,27].

This study introduces a novel approach to close the carbon cycle and enable the conversion of CO_2_ into fuels or chemicals. The proposed method involves utilizing pure formic acid generated from CO_2_RR as the microbial feedstock (Fig. 5d). It is worth noting that no bioconversion of formate was observed when a high salinity catholyte was used as the microbial feedstock, despite having the same composition in the final medium and adjusting the initial pH to 7.

### 3.7 CO_2_RR mechanism and isotope tracer of microbial formic acid conversion

**Fig. 6:**
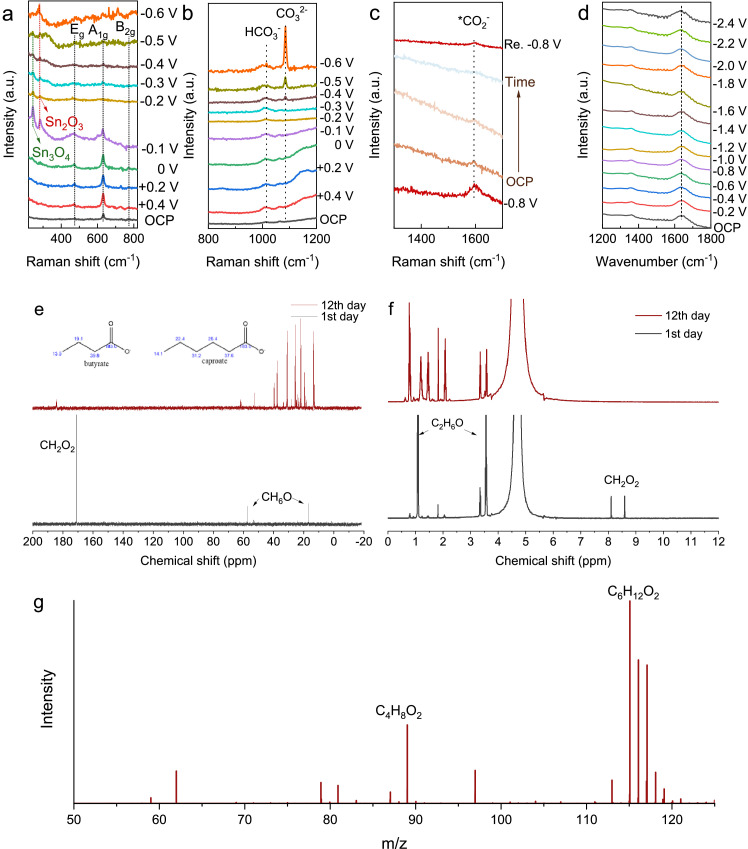
**CO_2_RR mechanism studies and isotope tracer results.** (a) *In-situ* electrochemical Raman for SnO_2_ electrocatalysts. (b) *In-situ* electrochemical Raman for the characterization of localized alkaline environment. (c) *In-situ* time mapping results. After OCP operation, the potential was re-fixed at −0.8 V for further verification. (d) *In-situ* ATR-IR spectra. (e) ^13^C-NMR (insets are the estimated results using ChemDraw). (f) ^1^H-NMR. (g) Mass spectrometry of samples at 12th day.

The *in-situ* electrochemical Raman spectroscopy was employed to elucidate the mechanism of CO_2_RR (Fig. 6a). The obtained spectra revealed distinct peaks at 472, 632, and 773 cm^−1^, corresponding to the E_g_, A_1g_, and B_2g_ vibration mode of SnO_2_ [44]. Notably, upon shifting the potential negatively to −0.1 V (vs. RHE), the intensity of these peaks rapidly decreased, accompanied by the emergence of peaks at 248 cm^−1^ and 288 cm^−1^, attributed to Sn_3_O_4_ and Sn_2_O_3_, respectively. Further negative potential shifts led to a gradual reduction of these peaks, indicating the conversion of oxidized Sn species to metallic Sn. Concurrently, the electrocatalyst surface experienced a shift towards a more alkaline microenvironment, as evidenced by increased peaks associated with CO_3_^2−^ (Fig. 6b).

In the t-mapping analysis of SHINERS (Fig. 6c), a peak at 1600 cm^−1^ was initially detected at a fixed potential of −0.8 V (vs. RHE). This peak exhibited a gradual decrease and nearly complete disappearance over the OCP duration, reappearing upon reapplication of the potential at −0.8 V. Notably, this potential-dependent peak at 1600 cm^−1^ was distinct from the G band [45], and was more likely associated with adsorbed *CO_2_^−^, favoring the formation of HCOO^−^ over CO [46].

A singular peak at 1630 cm^−1^ was identified in the *in-situ* ATR-IR spectra (Fig. 6d), likely associated with both H_2_O and the CO_2_RR intermediate *COOH [47]. Notably, the absence of the peak at 1400 cm^−1^, designated for the pivotal intermediate *OCHO crucial for formate formation, persisted even under high reduction potentials. This phenomenon aligns with observations from a prior investigation utilizing pure SnO_2_ for CO_2_RR, where achieving a partial current density exceeding 100 mA cm^−2^ proved challenging [47].

When utilizing formic acid-¹³C as the electron acceptor for the generation of MCFAs, the detection of butyric acid and caproic acid occurred within a mere 6 days when employing pre-enriched microbes, as confirmed by GC. Furthermore, ^13^C-NMR (Fig. 6e), ^1^H-NMR (Fig. 6f), and mass spectrometry (Fig. 6g) collectively substantiated the utilization of formic acid in the bioconversion step for chain elongation. To summarize, the investigation delved into the mechanism of the CO_2_RR, prompting the exploration of additional strategies to enhance the activity and stability of electrocatalysts. Certainly, the pure formic acid obtained from CO_2_ electroreduction was indeed utilized in product upgrading through microbial conversion.

### 3.8 Energy efficiency calculation and performance comparison

In this proof-of-concept study, a flow cell configuration was employed to maintain a relatively stable environment for the cathode catalyst. However, it's crucial to acknowledge that the large cathode-anode gap resulted in a significant ohmic drop, leading to cell voltages typically exceeding 6 V during full cell measurements (Fig. 2). Notably, previous studies on flow cell-based electrolysers have also reported cell voltages as high as 10 V [45]. To enhance efficiency and performance, a potential solution is to transition to a solid-electrolyte CO_2_ electrolyser with a MEA configuration. This switch increased the overall energy efficiency of power-to-formic acid production (considering all chambers) from approximately 12% to around 27%, primarily attributed to the reduction of ohmic drop (Fig. S18). In the bioconversion step, energy efficiency was calculated for different outputs: biomethane had an efficiency of 89.0 ± 0.2%, acetate had an efficiency of 76.6 ± 2.8%, MCFAs had an efficiency of 27.3 ± 0.9%, and bioelectricity had an efficiency of 26.3 ± 0.1%. These calculations facilitated the determination of the overall energy efficiency of the biohybrid CO_2_ electrolysis. For instance, with MEA-based solid-electrolyte CO_2_ electrolysers, the overall energy efficiency of power-to-formic acid-to-methane was approximately 24%, and the overall energy efficiency of the power-to-formic acid-to-bioelectricity process was approximately 7.1%. This aligns with previous studies on rechargeable microbial electrochemical systems using cube-shaped reactors (ranging from 0.6% to 4.5% efficiency) [48] or bottle-based reactors (ranging from 0.06% to 2.2% efficiency) [49]. It is imperative to implement strategies aimed at enhancing overall energy efficiency. Nevertheless, this study offers a promising approach for a closed carbon cycle, building upon previous reports focusing on the direct formate fuel cell [23], acetate-medicated bacteria–photocatalyst [26], and bi-directional hydrogenation of CO_2_ to formic acid [50].

To comprehensively understand the impressive performance achieved through external mode biohybrid CO_2_ electrolysis, various aspects were thoroughly examined and compared with those of representative prior studies (Table S2). In general, biohybrid CO_2_ electrolysis under external mode resulted in high current density, electron recovery, and energy recovery, while utilizing low-cost and commercially available SnO_2_ nanoparticles. Although CO_2_ electrolysers equipped with GDE but without SPC exhibited significantly higher current density [51], a drawback was the high energy consumption required for separating the products from the electrolyte [21,52]. It's important to note that just because CO_2_ electrolysis for formic acid production is more technologically advanced or ready [53,54], it doesn't diminish the significance or interest in studying CO_2_ supplied to microbial electrosynthesis. For detailed discussions on this point, several comprehensive review articles are recommended for reading [12,15].

## 4 Environmental implication

This study demonstrated the potential of biohybrid CO_2_ electrolysis under external mode to efficiently close the carbon cycle and convert CO_2_ into fuels or chemicals, specifically by producing pure formic acid as an intermediate. Notably, conventional studies on biohybrid CO_2_ electrolysis were predominantly conducted under internal mode, where intermediates were dissolved in a high concentration of electrolyte, resulting in current densities generally below 2 mA cm^−2^. In contrast, our study achieved a substantial increase in current density by an order of magnitude, reaching 60 mA cm^−2^.

The cell configuration can be optimized further to reduce energy consumption and enhance the stability of the GDE. Our findings indicate that the SPC packed in the extraction chamber contributes significantly to energy loss. Successfully reducing the thickness of the SPC from centimeter-scale to millimeter-scale using a MEA-based configuration was achieved. To enhance the concentration and Faradic efficiency of pure formic acid in the extraction chamber, a couple of straightforward methods were explored. First, increasing the volume ratio of the extraction chamber to the cathode chamber was considered (Fig. S19). Additionally, introducing a stationary layer of KOH solution between the AEM and GDE was evaluated to enhance formic acid recovery in the extraction chamber (Fig. S20). However, eliminating the cathode chamber and catholyte, as mentioned in some references [40], proved impractical due to increased resistance from water loss between the AEM and GDE (Fig. S21). Although hot pressing the AEM on the GDE reduced the internal resistor, it also led to a decrease in the Faradic efficiency of formate (Fig. S22 and Fig. S23).

In this study, no inhibition was observed when the collected formic acid solution, containing a maximum of 1.09 ± 0.02 mg L^−1^ of tin, was used as the microbial feedstock (Fig. 3). However, tin-based catalysts with multiple valence states can result in low Faradic efficiency during the CO_2_RR. The transition from polycrystalline SnO_2_ to polycrystalline Sn observed aligns with earlier reports [23]. Furthermore, the Faradic efficiency towards formic acid showed a decrease from approximately 80% to around 30% after operating for 20 h. Stability issues of the carbon-based GDE, a commonly encountered problem, were attributed to the loss of hydrophobic properties in the gas diffusion layer during operation [54]. To further enhance stability, engineering methods such as the development of alternative tin-based catalysts and/or the fabrication of GDE using polytetrafluoroethylene (PTFE) membranes should be considered [37].

Efforts should be directed towards minimizing carbon losses during microbial metabolism. While the utilization of formic acid in microbial metabolism is recognized as a promising method for efficient renewable energy storage [43], this study provides experimental evidence that a significant portion (three-quarters) of electro-reduced CO_2_ is released during microbial metabolism due to the utilization of lightly reduced formic acid as an intermediate. It's also essential to note that when targeting higher-value products, the issue of carbon loss during CO_2_RR in alkaline conditions can become more pronounced [55].

In this proof-of-concept study, communities were enriched when pure formic acid derived from CO_2_RR was used as the feedstock for generating bioelectricity, biomethane, and MCFAs (Fig. S24 and Fig. S25). The potential applications of this system can be expanded even further with the development of novel electrocatalysts and/or microbial catalysts. For instance, leveraging advanced electrocatalysts, the C_2+_ intermediates produced through CO_2_RR can be utilized in the subsequent bioconversion step [56,57]. Moreover, with significant advancements in microbial metabolic engineering, other valuable chemicals can be generated in this hybrid system.

## 5 Conclusion

This study introduces a biohybrid CO_2_ electrolysis operating under external mode, designed to produce various bio-based products, including bioelectricity, biomethane, acetate, and MCFAs, utilizing formic acid as an intermediate. The successful achievement of a high current density ensures the efficient production of pure formic acid during the CO_2_RR step. Furthermore, the subsequent bioconversion step demonstrated a remarkable level of selectivity. These results not only provide the first experimental evidence supporting the advantages of biohybrid CO_2_ electrolysis but also present a compelling approach to closing the carbon cycle and converting CO_2_ into valuable fuels or chemicals. Future endeavors should prioritize the reduction of energy consumption, improvement of electrocatalyst activity and stability, minimization of carbon losses, and the exploration of broader applications for this innovative technology.

## Declaration of competing interest

The authors declare that they have no conflicts of interest in this work.

## Appendix Supplementary materials

Supplementary materials Supplementary material associated with this article can be found, in the online version, at doi: Image, application 1
