# Supplementary material for: Biohybrid CO2 electrolysis under external mode: Using pure formic acid extracted from CO2 electroreduction for diverse microbial conversion
Source: Fundam Res. 2024 Mar 5;5(6):2597–606. doi: 10.1016/j.fmre.2024.02.008 (PMC12744634; doi:10.1016/j.fmre.2024.02.008)
Supplement: Supplementary file 1 — Supplementary materials Supplementary material associated with this article can be found, in the online version, at doi: [file mmc1.docx]

**Supplementary materials for**

**Biohybrid CO_2_ electrolysis** **under** **external mode: using pure formic acid extracted from CO_2_ electroreduction for** **diverse microbial conversion**

Na Chu ^a, b, c^, Xiaobing Wu ^c^, Ziyue Zhao ^d^, Xue Zheng ^c^, Yilin Lu ^e^, Ying Pu ^c^, Yue Wang ^c^, Jiayi Cai ^c^, Lixia Zhang ^a^, Xiaohong He ^a^, Daping Li ^a^, Raymond Jianxiong Zeng ^c^, Yangyang Yu ^e^, Yong Jiang ^c*^

^a^ CAS Key Laboratory of Environmental and Applied Microbiology, Environmental Microbiology Key Laboratory of Sichuan Province, Chengdu Institute of Biology, Chinese Academy of Sciences, Chengdu 610041, China

^b^ University of Chinese Academy of Sciences, Beijing 100049, China

^c^ Fujian Provincial Key Laboratory of Soil Environmental Health and Regulation, College of Resources and Environment, Fujian Agriculture and Forestry University, Fuzhou 350002, China

^d^ School of Life Science, Beijing Institute of Technology, Beijing 100081, China

^e^ Information Materials and Intelligent Sensing Laboratory of Anhui Province, Institutes of Physical Science and Information Technology, Anhui University, Hefei 230601, China

*Corresponding author (Yong Jiang), E-mail: jiangyongchange@163.com.

**There are 33 pages in SI, including 1 Text, 25 figures, and 2 tables.**

**Text S1 Equations for the calculation of efficiency**

Faradic efficiency of CO_2_ electrolysers and Coulombic efficiency for bioelectricity were calculated considering a certain molar conversion factor, according to eqn. (1), and eqn. (2), respectively.^[2]^ The energy efficiency of CO_2_ electrolysers (eqn. (3)) and energy efficiency for bioelectricity generation (eqn. (4)) were calculated, respectively. To perform the calculation of energy efficiency of chemicals production, the standard heat of combustion of chemical substances were used for formic acid (-254 kJ/mol), methane (-891 kJ/mol), acetate (-874 kJ/mol), ethanol (-1367 kJ/mol), and caproate (-3491.7 kJ/mol), respectively (eqn. (5)) ^[3]^. Please note that the evaluation conducted here focused on the full-cell energy efficiency rather than the half-cell energy efficiency.

$Faradic efficiency=\frac{Fb_{es}V\Delta c}{M_{s}\int_{0}^{t} I dt}$ (1)

where *Δc* is the product concentration change, *I* is the current consumption of CO_2_ electrolysers during the reaction time *t*, *M*_s_ is the molecular weight of the product, *F* is Faraday’s constant, *b*_es_ is the number of electrons exchanged per mole of product, and *V* is the volume of electrolyte used.

$Coulombic efficiency=\frac{M_{s}\int_{0}^{t} I dt}{Fb_{es}V\Delta c}$ (2)

where *Δc* is the substrate concentration change, *I* is the current output of microbial electrochemical reactors during the reaction time *t*, *M*_s_ is the molecular weight of the substrate, *F* is Faraday’s constant, *b*_es_ is the number of electrons exchanged per mole of substrate, and *V* is the volume of microbial electrochemical reactors.

$energy efficiency of CO_{2}RR=\frac{\Delta Hn_{p}}{\int_{0}^{t} EI\mathrm{dt}}$ (3)

where *ΔH* is the standard heat of combustion of formate (254 kJ mol^-1^), *n*_p_ is the amount (mol) of product generated in CO_2_ electrolysers during the reaction time *t*, *E* is the cell voltage, amd *I* is the current consumption of CO_2_ electrolysers.

$energy efficiency for bioelectricity generation=\frac{\int_{0}^{t} EI\mathrm{dt}}{\Delta Hn_{s}}$ (4)

where *ΔH* is the standard heat of combustion of formate, *n*_s_ is the amount (mol) of substrate consumed in microbial electrochemical reactors during the reaction time *t*, *E* is the cell voltage, and *I* is the current output of microbial electrochemical reactors.

$energy efficiency for chemicals production=\frac{{\Delta H}_{P}n_{p}}{{\Delta H}_{s}n_{s}}$ (5)

where *ΔH_s_* is the standard heat of combustion of substrates, *n*_s_ is the amount (mol) of substrates consumed, *ΔH_p_* is the standard heat of combustion of products, *n*_p_ is the amount (mol) of products generated.

⑥

⑤

④

①

②

③

**Figure S1.** GDE and SPC equipped solid-electrolyte CO_2_ electrolysers (four-chamber flow-cell) was used for CO_2_ electroreduction. (a) Schematic diagram, (b) photograph, (c) the detailed process for the assembly of the flow-cell, and (d) digital pictures displayed from an exploded view. The numbers marked showed the layer-by-layer steps. The SPC was added in the third step. Degreasing cotton was used in the inlet and outlet of the extraction chamber to ensure that the SPC particles not be flowed out.

**Figure S2.** A membrane electrode assembly (MEA) based solid-electrolyte CO_2_ electrolysers (4 cm^2^) was constructed, to further increase the energy efficiency. (a) Photograph, and (b) exploded view diagrams. The MEA was operated at 4 V. Same materials were used for the anode, cathode, and membrane, according to the construction of the flow cell based solid-electrolyte CO_2_ electrolysers. The flow rate of deionized water was fixed at 0.26 mL min^−1^, while the anolyte and catholyte were all fixed at 2 mL min^−1^. Deionized water was pumped into the extraction chamber in single-pass mode. The volume of the anolyte and catholyte were 100 mL and 10 mL, respectively.


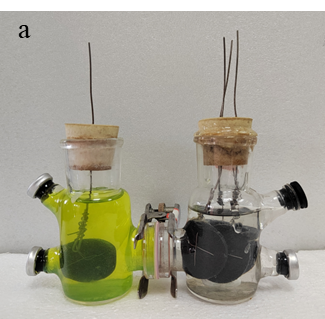


**Figure S3.** The preparation of anodic biofilms using a two-chamber MFC in our laboratory. (a) The photo of two-chamber MFC used for the preparation of anodic biofilms. (b) A typical cycle of cell voltage output of two-chamber MFC (results of two MFC reactors were presented). The anodic chamber was inoculated using the effluent from an MFC reactor fed with acetate in our laboratory ^[1]^. The composition of medium in the anodic chamber was: formic acid, ~20 mM; K_2_HPO_4_, 2.6 g L^-1^; KH_2_PO_4_, 4.4 g L^-1^; NH_4_Cl, 0.31 g L^-1^; MgCl_2_•6H_2_O, 0.2 g L^-1^; Na_2_SO_4_, 0.05 g L^-1^; trace elements, 12.5 mL; and vitamin solution, 5 mL. Composition of trace elements and vitamin solution were reported in previous study ^[2]^. Composition of medium in the cathodic chamber was: 16.64 g K_3_Fe(CN)_6_, K_2_HPO_4_, 2.6 g L^-1^; KH_2_PO_4_, 4.4 g L^-1^. Three pieces of circular carbon felt (diameter 2.2 cm, thickness 0.5 cm) were used in the two-chamber MFC for the preparation of anodic biofilms.

**Figure S4.** Performance of the adaptation period for MCFAs production, when feeding with formic acid and ethanol as the electron accepter and electron donor, respectively. Time course of (a) identified chemicals, and (b) pH of the medium. Formic acid and ethanol were replenished in the 16^th^ day, while the whole medium was refreshed at the 69^th^ day. The adaptation period was processed using medium: K_2_HPO_4_, 2.6 g L^-1^; KH_2_PO_4_, 4.4 g L^-1^; NH_4_Cl, 0.31 g L^-1^; MgCl_2_•6H_2_O, 0.2 g L^-1^; NaHCO_3_, 4.2 g L^-1^; Na_2_SO_4_, 0.05 g L^-1^; 2-bromoethanesulfonate (2-BES, inhibitor for methanogenesis), 1.0 g L^-1^; yeast extract, 0.05 g L^-1^. In addition, the molar ratio of formic acid: ethanol was selected as 1:4, while the total carbon content was fixed at 400 mM C.

**Figure S5.** Time course of pH when different current densities were applied to the GDE and SPC equipped four-chamber flow cell. Abbreviation: an., anode; ex., extraction; and ca., cathode. Marked numbers were current densities.

**Figure S6.** Effects of flow rates of deionized water in the extraction chamber on CO_2_ electroreduction. (a) Formic acid concentration, (b) Faradic efficiency of formic acid, (c) cathode potential, and (d) cell voltage. Abbreviation: an., anode; ex., extraction; and ca., cathode. Marked numbers were flow rates of deionized water in the extraction chamber.

**Figure S7.** Time course of pH when different flow rates of deionized water in the extraction chamber were adjusted in the GDE and SPC equipped four-chamber flow cell. Abbreviation: an., anode; ex., extraction; and ca., cathode. Marked numbers were flow rates of deionized water in the extraction chamber.

**Figure S8.** Performance of a traditional GDE equipped three-chamber flow-cell for CO_2_ reduction at a current density of 60 mA cm^-2^. (a) Cell voltage, and (b) formic acid concentration. Neither a solid-electrolyte nor an extraction chamber were utilized.

**Figure S9.** Performance of a GDE equipped four-chamber flow-cell without SPC for CO_2_ reduction at a current density of 60 mA cm^-2^. (a) Cell voltage, (b) cathode potential, (c) formic acid concentration, and (d) pH of solutions in different chambers. In the extraction chamber SPC was not packed but 1 M KOH was used.


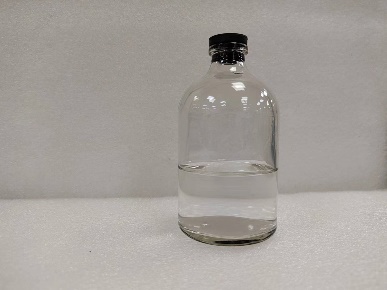


**Figure S10.** Transparency formic acid solution produced from CO_2_ electroreduction.

**Figure S11.** CV curves over a range of scan rates for used SnO_2_/C GDE after electroreduction.


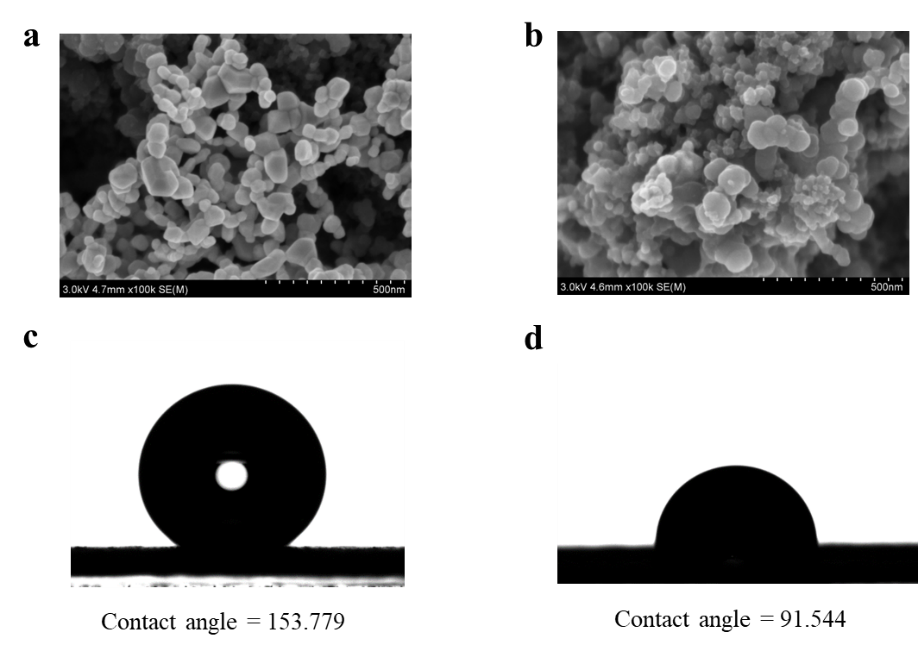


**Figure S12.** SEM of SnO_2_/C GDE (a) before and (b) after the CO_2_ electroreduction. Contact angle of SnO_2_/C GDE (c) before and (d) after the CO_2_ electroreduction. Current density was fixed at 60 mA cm^−2^ for CO_2_ electroreduction.

**Figure S13.** The long-term stability of the Bi/C GDE was evaluated using MEA based solid-electrolyte CO_2_ electrolysers. The MEA was operated at 4 V.

**Figure S14.** Bioelectricity generation in microbial 3-electrode setups when the formic acid-contained medium was prepared using analytical reagents.

**Figure S15.** Time course of biomethane generation using pure formic acid generated from CO_2_ electroreduction. (a) Substrates consumption and products generation, (b) pH, and (c) carbon distribution.

**Figure S16.** MCFAs generation using pure formic acid generated from CO_2_ electroreduction. The substrates consumption and products generation in (a) FA group feeding with only formic acid, and (b) ET group feeding with only ethanol. (c) Time course of pH during the bioconversion step.

**Figure S17.** Performance of MCFAs generation when using formic acid-¹³C as the electron acceptor. (a) Pre-enrichment of microbes using formic acid-^12^C and ethanol. (b) Chemicals generation detected by GC during 12 days’ bioconversion when using pre-enriched microbes and feeding with formic acid-^13^C and ethanol.

**Figure S18.** Energy efficiency of the (a) flow cell and (b) MEA based solid-electrolyte CO_2_ electrolysers, as well as (c) bioconversion step. Note that it is meaningless to calculated the energy efficiency of the non-spontaneous microbial 3-electrode setups, due to the lack of a cathode. So, the energy efficiency of bioelectricity generation from formic acid was calculated based on the data of Figure S3b. To perform the calculation, the standard heat of combustion of chemical substances were used for formic acid (-254 kJ/mol), methane (-891 kJ/mol), acetate (-874 kJ/mol), ethanol (-1367 kJ/mol), and caproate (-3491.7 kJ/mol), respectively ^[3]^.

**
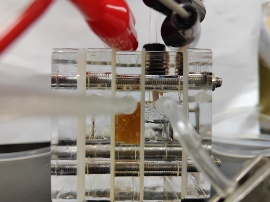
**

**Figure S19.** The concentration and Faradic efficiency of pure formic acid in the extraction chamber could be increased by simply increase the volume ratio of extraction chamber and cathode chamber. (a) Cell voltage, (b) concentration of formic acid, (c) Faradic efficiency of formic acid, and (d) pH. Current of 120 mA was applied to the GDE of 2 cm^2^. Here, the volume of the catholyte was decreased to 4 mL. After 3h’s operation, the concentration and Faradic efficiency of pure formic acid in the extraction chamber were reached to 2.9 g L^-1^ and 47.2%, respectively. The concentration and Faradic efficiency of formic acid/formate in the catholyte were reached to 4.2 g L^-1^ and 5.4%, respectively. The system was not stable. For example, the liquid level in the cathode chamber was decreased (inset figure), which could introduce the fluctuation of voltage. In addition, the pH of the cathode solution was reduced from its initial value of 13.67 to 9.15. The decreased pH could suppress the activity of SnO_2_ based GDE.

**Figure S20.** It was not stable using a stationary KOH solution layer between AEM and GDE. (a) Cell voltage and (b) Faradic efficiency. Based on the results of Figure S19, a stationary KOH solution layer (1 mm thickness，inset figure) was used between AEM and GDE, to further increase the volume ratio of extraction chamber and cathode chamber. Faradic efficiency of pure formic acid in the extraction chamber reached to 34.1% at 60 mA cm^-2^. However, the cell voltage dramatically increased and exceed 15 V after 17-23 min, depending on the current density. After disassembling the reactor, it was found that the moist porous support layer became dry, which was in agreement with that of Figure S19. These results indicated that water lost was occurred between AEM and GDE and the water management was important to further optimization the cell configuration of CO_2_ electrolysers.

**Figure S21.** A high cell voltage was required when the catholyte was omitted. Cell voltage and current with AEM cling to GDE. The applied 120 mA of current could not be applied to the CO_2_ electrolysers because of that the cell voltage exceed 15 V, which is the upper limit of the battery testing system (CT-4008; Neware Technology Co., Ltd., Shenzhen, China) used as the power source. Water lost could be occurred between AEM and GDE, which greatly increase the resistance of the CO_2_ electrolysers.


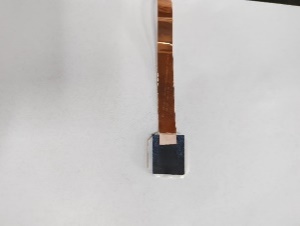

**Figure S22.** Hot pressing of AEM on the GDE successfully decreased the internal resistor, whereas, it also decreased the Faradic efficiency of formate. (a) Cell voltage and (b) formate generation performance after hot pressing of AEM on the GDE. (c) Cell voltage and (d) formate generation performance after hot pressing of AEM on the GDE (using 1.5 times of Nafion ionomer solution when airbrushing the SnO_2_/C GDE). Inset is a photo of hot pressing AEM on the GDE.

**Figure S23.** Validation that the hot pressing of AEM on the GDE decreased the Faradic efficiency of formate, by using conventional GDE equipped three-chamber flow cell, where a piece of anion exchange membrane (AEM) was used as the separator. (a) Cell voltage and (b) Faradic efficiency. The anolyte and catholyte was prepared using 1 M KOH. Faradic efficiency of formate was 5.2%.

**Figure S24.** Bacterial community at the (a) phylum level, and (b) genus level. Sequences less than 2% were grouped as “others”. The relative abundance was the average of two samples from duplicate reactors.

**Figure S25.** Archaea analysis at the phylum level and genus level. Sequences less than 1% were grouped as “others”.

**Table S1** Other impurity ions in the extraction solution at different flow rates.

| Flow rate (ml min^-1^) | K  (mg L^-1^) | Na  (mg L^-1^) | Fe  (mg L^-1^) | Bi  (mg L^-1^) | Ag  (mg L^-1^) |
| --- | --- | --- | --- | --- | --- |
| 0.5  2  10 | 6.76 ± 0.23  6.90 ± 0.05  7.82 ± 0.03 | 0.84 ± 0.11  1.08 ± 0.38  0.74 ± 0.12 | 0.12 ± 0.01  0.13 ± 0.03  0.12 ± 0.01 | 0.19 ± 0.02  0.19 ± 0.01  0.20 ± 0.01 | 0.04 ± 0.01  0.04 ± 0.01  0.04 ± 0.01 |

**Table S2** Summary of CO_2_ electrolysis and biohybrid CO_2_ electrolysis.

| Power to X | Configuration | Electrocatalysts | Microbial catalysts | Current  (mA cm^-2^) | Stability  (h) | Electron recovery (%) | Energy efficiency (%) | Energy consumption (kWh/kg X) | Ref. | |
| --- | --- | --- | --- | --- | --- | --- | --- | --- | --- | --- |
| Formate | Flow cell-SPC | SnO_2_ | N. A. | 60 | N. A. | 81.4 | 12 | 12.8 | **This study** |  |
|  | MEA-SPC | SnO_2_ | N. A. | 50 | 10 | 75 | 27 | 5.7 | **This study** |  |
|  | H-type | CuOx300 | N. A. | 8.1 | 96 | 0.5 | N. A. | N. A. | ^[4]^ |  |
|  | INFORS vessel | indium | N. A. | ~1 | 42 | 12.9 | N. A. | N. A. | ^[5]^ |  |
|  | H-type | CuOx | N. A. | ~2 | 24 | 0.8 | N. A. | N. A. | **^[6]^** |  |
|  | H-type | SnO_2_ | N. A. | ~0.2 | 1440 | ~75 | N. A. | N. A. | **^[7]^** |  |
|  | MEA-SPC | Bi_2_S_3_-derived | N. A. | 100 | 280 | 90 | N. A. | N. A. | **^[8]^** |  |
|  | MEA-SPC | In_2_O_3_@C | N. A. | 30 | 3 | 80 | N. A. | N. A. | **^[9]^** |  |
|  | Flow cell | In_2_O_3_@C | N. A. | ~170 | 9 | 93 | N. A. | N. A. | **^[9]^** |  |
| Methane | Flow cell-SPC | SnO_2_ | Mixed culture | 60 | N. A. | 81.4 | 10.7 | 144.6 | **This study** |  |
|  | MEA-SPC | SnO_2_ | Mixed culture | 50 | 10 | 75 | 24.0 | 64.3 | **This study** |  |
|  | Three-chambered cell | Tin | Methanococcus maripaludis | 2.66 | 100 | 33.3 | 8.3 | 186.2 | ^[10]^ |  |
|  | H-type | electroless-Cu | Mixed culture | 0.06 | 600 | 20 | N. A. | N. A. | ^[11]^ |  |
|  | H-type | Ag-Cu | N. A. | 8.3 | 2 | 60 | N. A. | N. A. | **^[12]^** |  |
|  | Flow cell | CuGa | N. A. | 149 | 1 | 51 | N. A. | N. A. | **^[13]^** |  |
|  | H-type | CuGa | N. A. | ~4.5 | 4 | 52 | N. A. | N. A. | **^[13]^** |  |
| Acetate | Flow cell-SPC | SnO_2_ | Mixed culture | 60 | N. A. | 73.9 | 9.2 | 44.0 | **This study** |  |
|  | MEA-SPC | SnO_2_ | Mixed culture | 50 | 10 | 68.1 | 20.7 | 19.6 | **This study** |  |
|  | H-type | Copper | Mixed culture | ~1.5 | 672 | 55 | N. A. | N. A. | **^[6]^** |  |
|  | H-type | N. A. | Mixed culture | 1.7 | 432 | 60 | 9.4 | 43 | ^[14]^ |  |
|  | H-type | Mo8@Cu/TNA | N. A. | 110 | 3 | 48.68 | N. A. | N. A. | **^[15]^** |  |
|  | Flow cell | PcCu-TFPN | N. A. | 12.5 | 80 | 90.3 | N. A. | N. A. | **^[16]^** |  |
|  | MEA-SSE | Ni–N–C single atom + GB-Cu | N. A. | 154 | 140 | ~37 | 6.52 | 62.1 | **^[17]^** |  |
| MCFA | Flow cell-SPC | SnO_2_ | Mixed culture | 60 | N. A. | N. A. | 3.3 | 253.0 | **This study** |  |
|  | MEA-SPC | SnO_2_ | Mixed culture | 50 | 10 | N. A. | 7.3 | 113.3 | **This study** |  |

**Reference:**

1. Chu N, Liang Q, Hao W, Jiang Y, Zeng RJ. Micro-microbial electrochemical sensor equipped with combined bioanode and biocathode for water biotoxicity monitoring. Bioresour Technol 2021, **326:** 124743.
2. Jiang Y, Liang P, Zhang C, Bian Y, Sun X, Zhang H, Yang X, Zhao F, Huang X. Periodic polarity reversal for stabilizing the pH in two-chamber microbial electrolysis cells. Appl Energy 2016, **165:** 670-675.
3. Haynes WM. *CRC handbook of chemistry and physics*. CRC press, 2016.
4. Chatzipanagiotou KR, Jourdin L, Buisman CJN, Strik DPBTB, Bitter JH. CO2 conversion by combining a copper electrocatalyst and wild‐type microorganisms. ChemCatChem 2020, **12**(15)**:** 3900-3912.
5. Hegner R, Neubert K, Kroner C, Holtmann D, Harnisch F. Coupled Electrochemical and Microbial Catalysis for the Production of Polymer Bricks. ChemSusChem 2020, **13**(19)**:** 5295-5300.
6. Chatzipanagiotou KR, Soekhoe V, Jourdin L, Buisman CJN, Bitter JH, Strik D. Catalytic cooperation between a copper oxide electrocatalyst and a microbial community for microbial electrosynthesis. CHEMPLUSCHEM 2021, **86**(5)**:** 763-777.
7. Gao T, Zhang H, Xu X, Teng J. Integrating microbial electrolysis cell based on electrochemical carbon dioxide reduction into anaerobic osmosis membrane reactor for biogas upgrading. Water Res 2021, **190:** 116679.
8. Lin L, He X, Zhang XG, Ma W, Zhang B, Wei D, Xie S, Zhang Q, Yi X, Wang Y. A Nanocomposite of Bismuth Clusters and Bi(2) O(2) CO(3) Sheets for Highly Efficient Electrocatalytic Reduction of CO(2) to Formate. Angew Chem Int Ed Engl 2023, **62**(3)**:** e202214959.
9. Wang Z, Zhou Y, Liu D, Qi R, Xia C, Li M, You B, Xia BY. Carbon-Confined Indium Oxides for Efficient Carbon Dioxide Reduction in a Solid-State Electrolyte Flow Cell. Angew Chem Int Ed Engl 2022, **61**(21)**:** e202200552.
10. Huang Y-X, Hu Z. An integrated electrochemical and biochemical system for sequential reduction of CO 2 to methane. Fuel 2018, **220:** 8-13.
11. Baek G, Shi L, Rossi R, Logan BE. Using copper-based biocathodes to improve carbon dioxide conversion efficiency into methane in microbial methanogenesis cells. Chem Eng J 2022, **435**.
12. Zhang H, Chang X, Chen JG, Goddard III WA, Xu B, Cheng M-J, Lu Q. Computational and experimental demonstrations of one-pot tandem catalysis for electrochemical carbon dioxide reduction to methane. Nat Commun 2019, **10**(1)**:** 3340.
13. Okatenko V, Loiudice A, Newton MA, Stoian DC, Blokhina A, Chen AN, Rossi K, Buonsanti R. Alloying as a Strategy to Boost the Stability of Copper Nanocatalysts during the Electrochemical CO(2) Reduction Reaction. J Am Chem Soc 2023, **145**(9)**:** 5370-5383.
14. Liang Q, Gao Y, Li Z, Cai J, Chu N, Hao W, Jiang Y, Zeng RJ. Electricity-driven ammonia oxidation and acetate production in microbial electrosynthesis systems. Frontiers of Environmental Science & Engineering 2022, **16**(4)**:** 42.
15. Zang D, Li Q, Dai G, Zeng M, Huang Y, Wei Y. Interface engineering of Mo8/Cu heterostructures toward highly selective electrochemical reduction of carbon dioxide into acetate. "Appl Catal, B " 2021, **281:** 119426.
16. Qiu XF, Huang JR, Yu C, Zhao ZH, Zhu HL, Ke Z, Liao PQ, Chen XM. A Stable and Conductive Covalent Organic Framework with Isolated Active Sites for Highly Selective Electroreduction of Carbon Dioxide to Acetate. Angew Chem Int Ed Engl 2022, **61**(36)**:** e202206470.
17. Zheng T, Zhang M, Wu L, Guo S, Liu X, Zhao J, Xue W, Li J, Liu C, Li X, Jiang Q, Bao J, Zeng J, Yu T, Xia C. Upcycling CO2 into energy-rich long-chain compounds via electrochemical and metabolic engineering. Nature Catalysis 2022, **5**(5)**:** 388-396.
